# The impact of the involvement of a healthcare professional on the usage of an eHealth platform: a retrospective observational COPD study

**DOI:** 10.1186/s12931-021-01685-0

**Published:** 2021-03-21

**Authors:** Cathelijne M. van Zelst, Marise J. Kasteleyn, Esther M. J. van Noort, Maureen P. M. H. Rutten - van Molken, Gert-Jan Braunstahl, Niels H. Chavannes, Johannes C. C. M. in ’t Veen

**Affiliations:** 1 Department of Pulmonology, Franciscus Gasthuis en Vlietland, Kleiweg 500, Rotterdam, 3045 PM The Netherlands; 2 Department of Pulmonology, Erasmus Medical Center, Rotterdam, The Netherlands; 3 Department of Public Health and Primary Care, Leiden University Medical Center, Leiden, The Netherlands; 4 National eHealth Living Lab, Leiden University Medical Center, Leiden, The Netherlands; 5 Department of Pulmonology, Leiden University Medical Center, Leiden, The Netherlands; 6 Erasmus School of Health Policy & Management, Erasmus University, Rotterdam, The Netherlands

**Keywords:** COPD, EHealth, CCQ, Adherence

## Abstract

**Background:**

Ehealth platforms, since the outbreak of COVID-19 more important than ever, can support self-management in patients with Chronic Obstructive Pulmonary Disease (COPD). The aim of this observational study is to explore the impact of healthcare professional involvement on the adherence of patients to an eHealth platform. We evaluated the usage of an eHealth platform by patients who used the platform individually compared with patients in a blended setting, where healthcare professionals were involved.

**Methods:**

In this observational cohort study, log data from September 2011 until January 2018 were extracted from the eHealth platform Curavista. Patients with COPD who completed at least one Clinical COPD Questionnaire (CCQ) were included for analyses (n = 299). In 57% (n = 171) of the patients, the eHealth platform was used in a blended setting, either in hospital (n = 128) or primary care (n = 29). To compare usage of the platform between patients who used the platform independently or with a healthcare professional, we applied propensity score matching and performed adjusted Poisson regression analysis on CCQ-submission rate.

**Results:**

Using the eHealth platform in a blended setting was associated with a 3.25 higher CCQ-submission rate compared to patients using the eHealth platform independently. Within the blended setting, the CCQ-submission rate was 1.83 higher in the hospital care group than in the primary care group.

**Conclusion:**

It is shown that COPD patients used the platform more frequently in a blended care setting compared to patients who used the eHealth platform independently, adjusted for age, sex and disease burden. Blended care seems essential for adherence to eHealth programs in COPD, which in turn may improve self-management.

## Introduction

Chronic Obstructive Pulmonary Disease (COPD) is a chronic airway disease characterized by an irreversible airway obstruction [1]. As one of the leading causes of chronic morbidity worldwide, it ranks fourth on the worldwide list of Disability Adjusted Life Years (DALY). To alleviate this burden, digital health support may be a potential solution.

Currently, non-pharmaceutical treatments reinforced by improved self-management skills, are key in treating COPD patients. Self-management strategies empower patients to change and influence their behavior to manage disease more effectively [1]. Based on a recent Delphi process, an international panel of COPD self-management experts published the following on self-management interventions: “A COPD self-management intervention is structured but personalized and often multi-component, with goals of motivating, engaging and supporting the patients to positively adapt their health behavior(s) and develop skills to better manage their disease” [2]. Self-management strategies lead to a significant increase in quality of life, six-minute walk test, self-efficacy, reduced duration of exacerbations and hospitalizations, and decreased healthcare costs [3–6]. Spruit et al. also discuss the critical role of behavioral change in pulmonary rehabilitation in chronic disease management. He described the major barrier to participation is accessibility [7]. Access may be limited by geography, finance, transport, culture and logistics. eHealth platforms may solve this problem. They can facilitate education of self-management on a large scale and may lead to behavioral change [8].

eHealth is an upcoming term in the Public Health sector, containing a set of different concepts, including health, technology and commerce [9]. Shaw et al. described three prominent but overlapping domains of eHealth: (1) monitoring, tracking and informing patients; (2) digital technologies used for communication between healthcare professional and patient; (3) collecting, managing and using health data [10]. An eHealth platform can be used by patients themselves on an individual basis or in a blended care setting. Using an eHealth platform in a blended care setting means online collaboration between patient and healthcare professional. Especially in the COPD population, mostly vulnerable, relatively old and often lower educated, this blended care setting may increase platform adherence. The impact of collaboration with a healthcare professional in self-management programs has been studied previously. In 2012, Fan et al. reported an increase in mortality in patients that pursued a comprehensive care management program (CCMP). The CCMP intervention consisted of individual weekly sessions using an educational booklet with an overview of COPD topics (e.g. respiratory symptoms and self-initiation of an antibiotic or prednisone for an exacerbation) with a call from a case manager once per month. It is discussed that the high mortality is possibly due to the lack of collaboration between the healthcare professional and the patient [11]. A quantitative study on perceptions and behaviors related to self-management diaries for asthma and COPD, showed positive effects on disease coping by regonizing exacerbations and adjusting medications. However, patients experienced practical barriers to integrating the diaries in their daily life [12]. Usage of a self-management programme in routine care can improve self-efficacy over time in COPD patients [13]. An increase in the use of a COPD eHealth platform in blended care setting was seen in a controlled study [14]. However, until now, no real-life data on this is available. It is necessary to achieve long-term eHealth platform adherence for COPD patients, to improve insight in their symptoms, and potentially improve their self-management abilities. To stimulate self-management in COPD patients, it is necessary that these patients recognize the severity of their symptoms. To achieve this, it is essential to observe their COPD symptoms for a longer period. More insight in symptom-fluctuation over time by closer monitoring of COPD patients improves patient empowerment and thus health status. There are different possibilities to observe COPD symptoms, such as measuring blood oxygen levels, observe forced expiratory volume in one second and by filling in the COPD questionnaires such as the Clinical COPD Questionnaire.

The aim of this observational study is to compare the usage of an eHealth platform between patients who use the platform in a blended setting to those who use it individually. We hypothesize that patients use the platform more frequently in the blended setting, which indicates an improved adherence.

## Methods

### Study design, setting, and participants

Real-life data of the eHealth platform Curavista (www.curavista.health) were used. Curavista is an open online certified (NEN7510, ISO 27001, CE class I MDD) eHealth platform. Currently, the platform hosts 80 different modules for managing health in chronic diseases, for example COPD, Diabetes Mellitus and Parkinson. Modules contain forms, questionnaires, e-consultations, monitoring and self-management programs. Individuals can use this platform independently or with the assistance of their healthcare professional. For the current study, data from September 2011 until January 2018 were extracted. In the current study analyses, we included all people who: (1) signed up for the COPD module (2) gave informed consent to analyze their data and (3) entered an age of ≥ 18 years old. People could delete their records at all times without giving a reason. Only patients that had at least one complete Clinical COPD Questionnaire (CCQ) on the Curavista platform were included for analyses. The CCQ is a questionnaire for COPD patients to score the severity of their symptoms [15]. The questionnaire consists of 10 items with 7 answer possibilities (0, no symptoms or no limitations; 6, severe symptoms or limitations). Total score ranges from 0–60, a higher score indicates a worse health status [16].

There were several ways patients could be notified about the platform. Firstly, patients were able to find the platform through advertising by the Lung Foundation Netherlands, and use the platform on their own without assistance of a healthcare professional. The Lung Foundation Netherlands is a large platform active in the Netherlands. On this platform patients can find information about pulmonary diseases and healthcare professionals can be informed about research projects. Further, volunteers of the Lung Foundation advertise for conventions and patient meetings. Secondly, patients could be invited by their healthcare professionals (i.e. a medical doctor or a respiratory nurse) in primary or hospital care and use the platform in a blended care setting. For all patients, with or without healthcare professional involved in the platform, it was possible to fill in a CCQ daily; they all received a reminder to do so every three months.

### Curavista COPD module

**Fig. 1:**
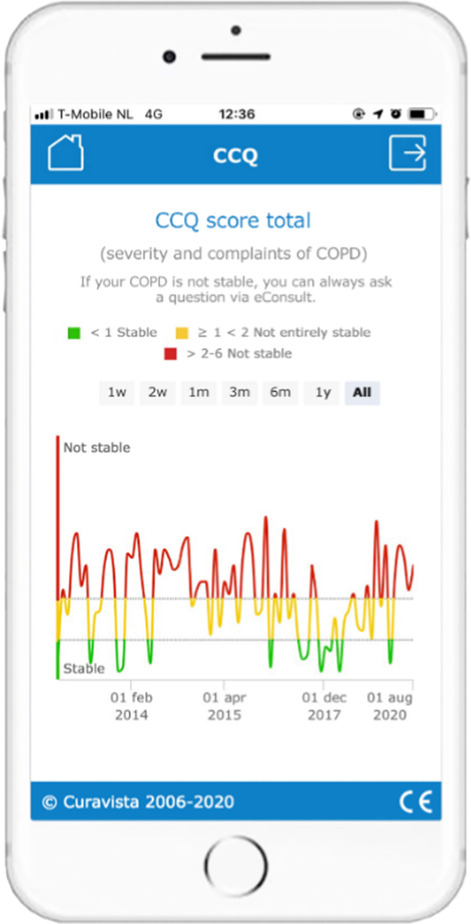
Example of a frequent submitter. CCQ scores > 2 is coloured red, orange included CCQ score 1 and 2 and green is a CCQ score < 1

**Fig. 2:**
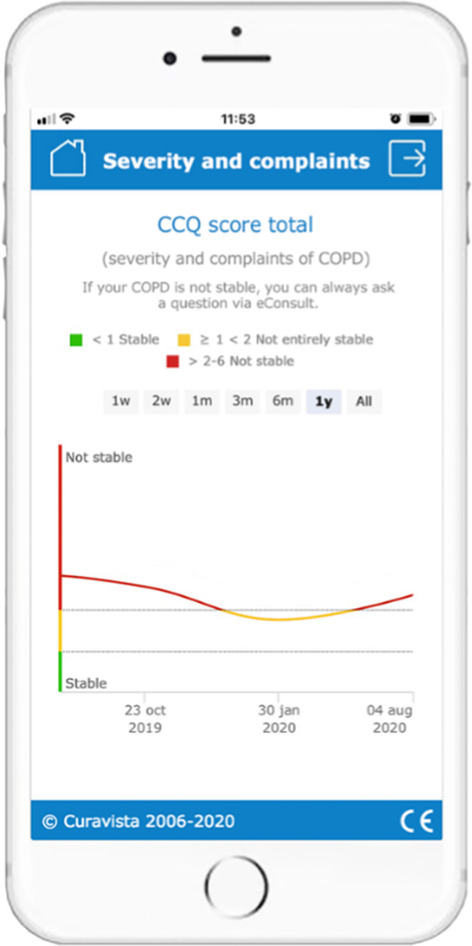
Example of a non-frequent submitter. CCQ scores > 2 is coloured red, orange included CCQ score 1 and 2 and green is a CCQ score < 1

The COPD module contains Patient Reported Outcome Measures (PROMs), including the Clinical COPD Questionnaire (CCQ), Medical Research Council Dyspnoea (MRC), Assessment of Burden of COPD (ABC)-tool, exacerbation plan, information about inhalation techniques and eConsult. This COPD module is validated in 2015 [12]. The COPD module was at first designed to be used in a blended care setting together with a healthcare professional, during consultation or for remote monitoring. Later, it became possible for people to use the system independently as well. The role of the healthcare professional in the blended care setting, in both primary and hospital care, is to check and discuss the results of the included PROM’s with the patient. The time in between these regular consults varies per individual. In general, a higher burden of disease leads to more consultations. The healthcare professionals are trained for motivational interviewing. Two examples of the platform are shown in Figs. 1 and 2; a frequent submitter and a non-frequent submitter. CCQ scores > 2 mean not stable and are colored red, orange included CCQ score 1 and 2 and is not entirely stable. Green is a CCQ score < 1 and refers to a stable COPD state. These cut-off points are commonly used in primary care [17].

### Data collection

All variables were extracted from the eHealth platform Curavista. CCQ submissions were used to operationalize usage of the platform. The date of the first CCQ-submission was used as starting point for using the eHealth platform. Data collection included age, sex, symptom scores at baseline and at the end of participation (first and last CCQ score) and participation length (number of days between date data extraction and date first CCQ-submission). The two groups of patients, i.e. patients who used the eHealth platform independently and those who used it in a blended care setting, naturally emerged from their registration. The outcome of the analysis was CCQ-submission rate as an indicator of the extent to which the eHealth platform was used. This research was declared as outside the scope of the Medical Research Involving Human Subjects Act. Patients’ informed consent was obtained to use their data for non-commercial anonymous analyses and they were able to delete their records at all times.

### Analyses

Patients whose data were incomplete were excluded from the analyses. Propensity score matching was used to reduce the bias in estimating the effect of CCQ-submission rates, in patients using the eHealth platform with or without healthcare professional. For this propensity score matching, a logistic regression was used. For this analysis, using the platform in a blended care setting was the dependent variable and the characterstics of the patient (age, sex and score of first CCQ-submission) were predictors. Probabilities were estimated, ranging from 0 to 1, for each patient in the study population. Due to the propensity score matching, n = 44 patients were excluded for analyses. For descriptive data (age, participation score of first CCQ submission and score of last CCQ submission), normally distributed continuous variables were reported as means with standard deviations, non-normally distributed continuous variables as medians with 25 and 75th interquartile ranges (IQRs) and categorical variables as numbers with percentages. Patients who used the platform with a healthcare professional were compared with patients who used the platform independently, using Chi-square test for categorical data, Mann Whitney-*U* tests for non-normally distributed continuous variables and unpaired *T*-test for normally distributed continuous variables. A *p*-value of < 0.05 was considered statistically significant.

Multivariate Poisson regression analyses were used to compare CCQ-submission rates between the group using the eHealth platform independently and the group using the platform in a blended care setting. Patients with two or more CCQ-submissions were included in these analyses. In the Poisson regression model, the group using the eHealth platform independently was used as reference group. Participating in a blended setting vs. independent use was included as an independent variable, CCQ-submission rate was used as dependent variable. The duration of participation (log-transformed) in days was used as offset variable. To reduce confounding, we included sex, age and CCQ score at first completion as covariates. Furthermore, we performed a second multivariate Poisson regression analysis to estimate the impact of type of healthcare setting (hospital vs. primary care setting) in the blended care group. In this analysis, the group using the eHealth platform in primary care was used as reference group. SPSS version 25.0 was used for all analyses.

## Results

**Fig. 3:**
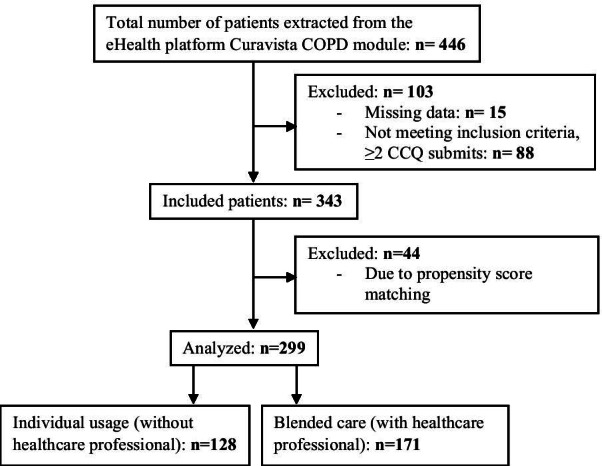
Flowchart of patient enrollment process

A total of 299 patients were included in this study; 57% (n = 171) used the platform in collaboration with a healthcare professional (“blended care group”) and 43% (n = 128) independently (“independent user group”). Healthcare professionals worked either in hospital (n = 142) or in primary care (n = 29). Missing patient data included age (n = 14) or sex (n = 1) (Fig. 3). The median [IQR] age of the patients in the blended care group and in the independent user group were comparable 65.8 [60.5–72.0] vs. 66.5 [58.9–73.2], p = 0.939, (Table 1).

**Table 1:** Characteristics of the study population at baseline after propensity score matching, with and without healthcare professional

	Without healthcare professional (n = 128)	With healthcare professional (n = 171)	*P*-value
Male N (%)	72 (56.3)	79 (46.2)	0.085
Age Y median [IQR]	66.5 [58.9–73.2]	65.8 [60.5–72.0]	0.939
Score of first CCQ submission [IQR]	4.0 [3.0–5.0]	3.0 [2.0–4.0]	< *0.001*
Score of last CCQ submission [IQR]	3.7 [2.2–5.0]	3.0 [2.0–4.1]	0.071

Median (IQR Q1–Q3) used in non-normal distribution *N* numbers, *IQR* interquartile range 25–75th percentile

**Fig. 4:**
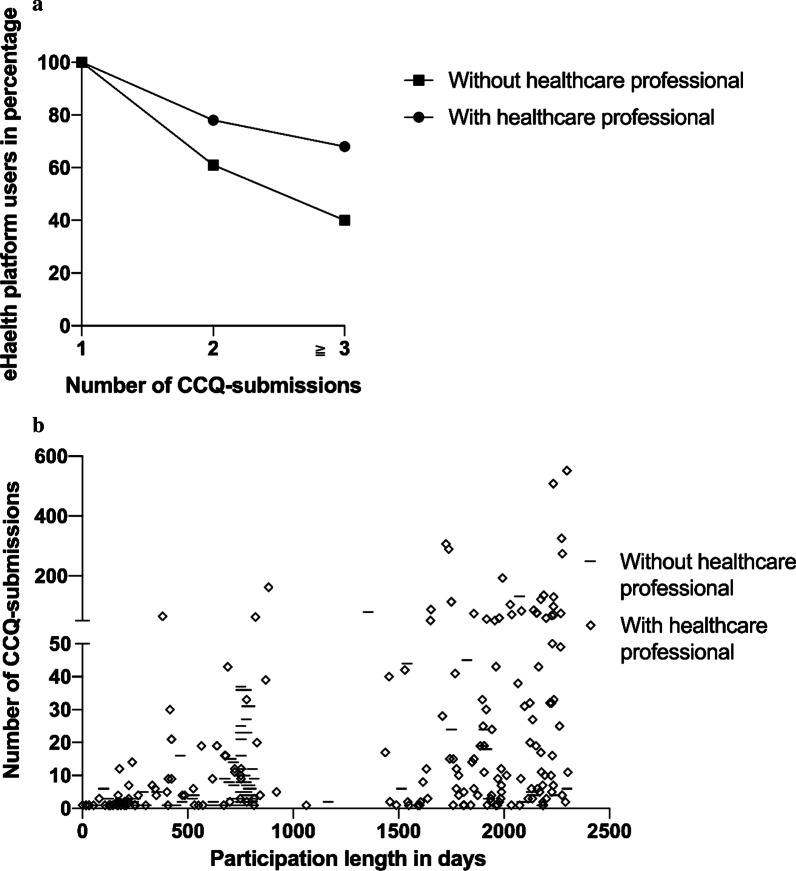
**a** Number of CCQ-submissions in users with and without healthcare professional. **b** Number of CCQ-submissions and participation length in days in users with and without healthcare professional

The median [IQR] scores of the first CCQ were significantly lower in patients in the blended care group compared to the patients in the independent user group [3.0 (2.0–4.0) vs. (4.0 (3.0–5.0), *p* < 0.001]. The last CCQ-submission showed a similar trend in patients in the blended care group vs. independent user group [3.0 (2.0–4.1) vs. 3.7 (2.2–5.0), *p* = 0.071]. The difference in number of CCQ submissions of the eHealth platform between the patients in the blended care group vs. independent user group is shown in Fig. 4a, b. In the group without healthcare professional 54% remained stable after intervention, where stable is defined as a change in CCQ between − 0.4 and 0.4. In the blended care group 45% remained stable (data not shown).

In total 211 patients were included in the Poisson analyses. In the crude Poisson analysis blended care was associated with a 3.00 (95% CI: 2.79–3.23, *p* < 0.001) higher CCQ-submission rate compared to patients who used the platform independent (Table 2). In the adjusted analysis, the CCQ-submission rate was 3.25 (95% CI: 3.01–3.50, *p* < 0.001) higher for patients in a blended care setting compared to patients who used the platform independently. The adjusted analyses of the blended care group showed a 1.83 (95% CI: 1.66–2.01, *p* < 0.001) higher CCQ-submission rate in the hospital care group compared to the primary care group.

**Table 2:** Difference in number of CCQ-submission rates

Model	Aim 1^a^		
	Rate; Exp (B)	95% CI	*p*-value
1^b^	3.001	2.792–3.226	< 0.001
2^c^	3.245	3.009–3.500	< 0.001

^a^Difference in number of CCQ-submission rates in patients participating independently or in a blended care setting (independently group ref)

^b^Crude (unadjusted) analysis

^c^Analysis adjusted for sex, age and score of first CCQ-submission

## Discussion

In this real-life study, we showed that COPD patients used an eHealth platform more frequent in a blended care setting compared to patients who used the platform independently, adjusted for sex, age and burden of disease. Our results imply that blended care results in higher usage of an eHealth platform. This confirms the crucial role of the healthcare provider in applying blended care and thus involving the patient in the e-program.

Our results confirm a prospective controlled study of Talboom-Kamp et al. [14], in which the visits of an eHealth platform among a COPD patient group with high assistance and low assistance in primary care were compared. The high-assistance group used the self-management platform statistically significant more frequent than the low-assistance group. We reported a higher usage of the eHealth platform in a blended care setting.

Literature on CCQ monitoring on blended care is not conclusive on the assumption that it improves the health status of COPD patients. An increase in mortality in a new comprehensive care management program has been shown, possibly due to the lack of collaboration between the healthcare professional and the patient [11]. In the field of psychology, improvement in adequate behavior is seen in a blended-care setting. For example, internet-based cognitive behavior therapy for symptoms of depression and anxiety was more effective with the support of a professional, than internet-based intervention without professional support [18]. A positive effect of blended care is also visible in the treatment of panic disorders: internet administrated self-help plus minimal therapist contact via e-mail had the same effect as traditional individual cognitive behavior therapy [19]. In pulmonology, an improvement of the subscale CCQ symptom score was found after participating in an eHealth platform for a prospective COPD study in a blended care setting [20]. In a meta-analysis about the effectiveness of eHealth intervention in Obstructive Sleep Apnea (OSAS) no difference was found between studies using eHealth as an add-on to care as usual and studies using eHealth as a replacement of care as usual [21]. The nightly use of CPAP (adherence) increased, with eHealth interventions compared to care as usual, regardless of the involvement of a healthcare professional. The different purposes of the eHealth platforms in blended care setting may explain the differences in outcomes. It is proven that nightly use of CPAP during three months improves self-reported sleepiness after four hours of use every night [22]. We speculate it is easier to achieve adherence when patients experience a direct effect on their symptoms. In this study, we did not have data to investigate if a better adherence to the eHealth platform led to changes in COPD (self-management), and whether that- in turn- led to changes in health status.

A strength of the study is taking the score of the first CCQ-submission into account, which makes it possible to conclude that patients, irrespective of disease burden, use the eHealth platform more frequently in a blended care setting. Secondly, to reduce bias, propensity score matching and adjustment on sex, age and first CCQ score were used in these analyses. These two methods are complementary and best used in combination. Third, the time between the first and last CCQ submission was taken into account, so we adjusted for a potential impact of length of participation on the results. Fourth, by using real-life data, we were able to generate results that are more likely to be externally valid. In Randomized Control Trials (RCTs) only a small and highly selected fraction of the real-life population is used, because of the strict inclusion criteria. Herland et al. [23] have presented that in case of strict COPD criteria (Obstructive lung disease and FEV1 < 70% of predicted normal, > 15 pack-years and absence of atopy), only 17% of the COPD population would be included in a clinical trial. Travers et al. [24] suggests only 5% of the COPD patients meet the inclusion criteria for major RCTs, which implies limited external validity. By using real-life data, this study emphasizes the conclusion of Talboom-Kamp et al. [14] in which the high-assistance group used the self-management platform significantly more frequent than the low-assistance group. Fifth, this study has evaluated the effectiveness of an eHealth platform, in contrast to most COPD eHealth platforms, which have not been evaluated [25].

One of the limitations of this study is the limited information available about outcomes in study participants. In addition, it was not possible to compare the submission per healthcare professional because of a lack of data. However, our main result is not affected by this limitation because using the eHealth platform with a healthcare professional, reveals higher CCQ-submission rates. An extraction date was used; at that time all data of the platform was downloaded for the analyses. If patients signed up close to the extraction date, they had had a shorter follow-up time and consequently less CCQs could have been submitted. Most presumably, this has a minor impact on the results as we included time as an offset in the Poisson analyses and patients were included in a time-range of seven years.

A second limitation of the study is that participants without a healthcare professional could potentially sign up for the COPD module without a COPD diagnosis of a pulmonologist or following the GOLD criteria [1]. However, because the eHealth platform was only offered to the general public via the website of the Lung Foundation Netherlands, it is unlikely participants without COPD would find this platform and submit at least two CCQs. The CCQ score of the patients without healthcare professional is higher, which indicates more complaints. Rennard [26] showed that only 15–20% of cigarette smokers visit a physician with symptoms. In the remaining 80–85% of the patients, lung function is usually abnormal. Thereby, in this study CCQ-submission rate was used as surrogate marker of usage of the eHealth platform. However, CCQ-submission rate might underestimate the actual usage of the platform, because other functions were not included such as usage of eConsult, submission of the MRC or usage of the knowledge base. Yet, it was possible to visit the platform without filling in a CCQ, but for example do an eConsult or submit the MRC questionnaire. A third limitation is: it was not possible to measure a direct (behavior) or indirect (QoL and exacerbations) effect on self-management, because of a lack of data before and after usage of the eHealth platform [27]. Thereby, an effect on the quality of life is dependent on different variables and possibly not an isolated effect of the eHealth platform. We can only suggest an improvement of self-management because of higher CCQ-submission rates and therefore adherence. Attrition, which is defined as participants stopping usage and/or being lost to follow up, is also an important topic in eHealth. This is important since many eHealth applications report high numbers of dropouts and non-users [28]. It is interesting to define the group in which this application works and to define the dropping out group. Because of a lack of data we were not able to look into attrition, but we expect in a blended care setting not only higher usage but also longer usage because of the guidance of a healthcare professional. A fourth limitation is the inability to make a distinction between submissions by mobile or by desktop. If patients who submitted in 2011 were more likely to be non-adherent, this effect can be explained by the absence of the technical improvements that made the eHealth platform more user-friendly in later years.

Filling in questionnaires and submitting does not directly have an effect on the COPD symptoms, which makes it hard to achieve improved adherence to an eHealth platform. We speculate it is more relevant for patients to submit CCQs when they know a healthcare professional is involved and can act on a severe score of the CCQ. Our study shows a positive effect on usage of an eHealth platform in a blended care setting, we believe that this might in turn have a positive effect on self-management.

## Conclusion

This study presents a higher usage of the eHealth platform in a blended care setting, adjusted for sex, age and disease burden. Blended care seems essential for adherence to eHealth programs in COPD and thereby may support self-management. Further research into the usage of eHealth platforms in a blended care setting is needed to demonstrate an improvement in self-management.

## Data Availability

The datasets used and/or analysed during the current study are available from the corresponding author on reasonable request.

## Abbreviations

COPD: Chronic Obstructive Pulmonary Disease
DALY: Disability Adjusted Life Years
HCP: Healthcare professional
CCQ: Clinical COPD Questionnaire
PROMs: Patient Reported Outcome Measures
MRC: Medical Research Counsil
QOL: Quality of Life
CPAP: Continuous Positive Airway Pressure
